# OpenMebius: An Open Source Software for Isotopically Nonstationary ^13^C-Based Metabolic Flux Analysis

**DOI:** 10.1155/2014/627014

**Published:** 2014-06-11

**Authors:** Shuichi Kajihata, Chikara Furusawa, Fumio Matsuda, Hiroshi Shimizu

**Affiliations:** ^1^Department of Bioinformatic Engineering, Graduate School of Information Science and Technology, Osaka University, 1-5 Yamadaoka, Suita, Osaka 565-0871, Japan; ^2^Quantitative Biology Center, RIKEN, 6-2-3 Furuedai, Suita, Osaka 565-0874, Japan

## Abstract

The *in vivo* measurement of metabolic flux by ^13^C-based metabolic flux analysis (^13^C-MFA) provides valuable information regarding cell physiology. Bioinformatics tools have been developed to estimate metabolic flux distributions from the results of tracer isotopic labeling experiments using a ^13^C-labeled carbon source. Metabolic flux is determined by nonlinear fitting of a metabolic model to the isotopic labeling enrichment of intracellular metabolites measured by mass spectrometry. Whereas ^13^C-MFA is conventionally performed under isotopically constant conditions, isotopically nonstationary ^13^C metabolic flux analysis (INST-^13^C-MFA) has recently been developed for flux analysis of cells with photosynthetic activity and cells at a quasi-steady metabolic state (e.g., primary cells or microorganisms under stationary phase). Here, the development of a novel open source software for INST-^13^C-MFA on the Windows platform is reported. OpenMebius (Open source software for Metabolic flux analysis) provides the function of autogenerating metabolic models for simulating isotopic labeling enrichment from a user-defined configuration worksheet. Analysis using simulated data demonstrated the applicability of OpenMebius for INST-^13^C-MFA. Confidence intervals determined by INST-^13^C-MFA were less than those determined by conventional methods, indicating the potential of INST-^13^C-MFA for precise metabolic flux analysis. OpenMebius is the open source software for the general application of INST-^13^C-MFA.

## 1. Introduction

The* in vivo* measurement of metabolic flux by ^13^C-based metabolic flux analysis (^13^C-MFA) provides valuable information regarding cell physiology in fields ranging from the metabolic engineering of microorganisms to the analysis of human metabolic diseases [1–3]. Since metabolic fluxes are estimated by a computational analysis of the isotopic labeling data produced by a series of wet experiments [4–7], the development of an open software platform for ^13^C-MFA is desired for further methodology improvement and wider applications for* in vivo* metabolic flux measurement.

**Figure 1:**
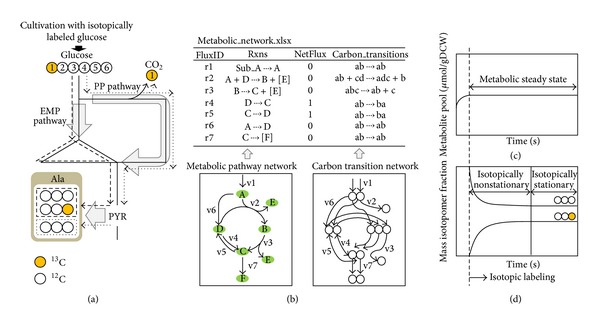
Principle of ^13^C-based metabolic flux analysis. (a) Principle of ^13^C-based metabolic flux analysis (^13^C-MFA). Isotopic enrichment of alanine depends on metabolic flux via the Embden-Meyerhof-Parnas (EMP) pathway or the pentose phosphate (PP) pathway. (b) The configuration of the model is described in “Metabolic_network.xlsx.” The metabolic reactions and the carbon transfer are described in the “Rxns” and “Carbon_transitions” columns, respectively. Detailed rules are provided in the tutorial on the project home page. ((c) and (d)) Metabolic steady state and isotopically stationary. The isotopic labeling experiment is performed under metabolic steady state. After feeding ^13^C-labeled glucose, isotopic labeling enrichment changes in a time-dependent manner and then reaches a stationary condition. Whereas cells are sampled under isotopically stationary conditions in conventional ^13^C-MFA, time courses of isotopic labeling enrichment during an isotopically transient state are used for INST-^13^C-MFA.

In ^13^C-MFA, after feeding of a ^13^C-labeled carbon source into a cell culture, amino acids or intermediates are extracted and subjected to mass spectrometric analysis. For the simplest example, [1-^13^C] glucose is converted to pyruvate (PYR) and then alanine (Ala) via two glycolytic pathways including the Embden-Meyerhof-Parnas (EMP) pathway and the pentose phosphate (PP) pathway (Figure 1(a)). Whereas one ^13^C-labeled molecule and one nonlabeled molecule of Ala are generated from one molecule of [1-^13^C] glucose by the EMP pathway, no ^13^C-labeled Ala is produced via the PP pathway, because the ^13^C atom is metabolically discarded as CO_2_. Thus, the metabolic flux ratio between the EMP and PP pathways could be estimated from the relative abundances of ^13^C-labeled and nonlabeled Ala using mass spectrometry.

In ^13^C-MFA of complex networks of carbon central metabolism, metabolic fluxes are computationally estimated by a nonlinear optimization method since the relationship between metabolic fluxes and isotopic labeling enrichment is usually nonlinear. For that purpose, a metabolic model *M* is constructed based on the metabolic pathway network and the carbon transition network, which represents the transitions of carbon atoms between substrates and products in a metabolic reaction (Figure 1(b)). *M* is a function to calculate isotopic labeling enrichment or the mass distribution vector (MDV) of metabolites from the given metabolic fluxes and isotopic labeling patterns of carbon sources. Consider

(1) $$\begin{array}{c} {\text{MDV}}_{j}^{\text{sim}}=M\left(v,x^{\text{inp}}\right). \end{array}$$

Here, MDV_*j*_^sim^ is a simulated mass spectrum of metabolite *j*. *v* and *x* ^inp^ are the vectors of metabolic flux and isotopic labeling pattern of carbon source, respectively. A vector of metabolic flux *v* is fitted to the observed mass spectrum (${\text{M}\hat{\text{D}}\text{V}}_{j}$) by a nonlinear optimization method:

(2) D=MD^Vj−MDVjsim,vopt=argminv∑j=1N(DTCMD^Vj−1D), s.t.  Sv=0.

The optimized value *v*_opt_ is the estimated metabolic flux distribution in the cells to minimize the covariance-weighted sum of squared difference. $C_{{\text{M}\hat{\text{D}}\text{V}}_{j}}$ is the covariance matrix with a measurement standard deviation located on the diagonal. *S* is the stoichiometric matrix. There are several software packages to perform conventional ^13^C-MFA such as 13CFLUX [8], 13CFLUX2 [9], C13 [10], Metran [11], FIA [12], influx_s [13], and OpenFLUX [14].

In the case of conventional ^13^C-MFA, isotopic labeling data must be obtained from cell culture under metabolic steady state and isotopically stationary conditions (Figures 1(c) and 1(d)). Here, metabolic steady state indicates the constant flux distribution and pool size of intracellular metabolites that has to be maintained during the isotopic labeling experiment (Figure 1(c)). An isotopically stationary condition means constant isotopic labeling enrichment of metabolites. A long culture period has often been required to achieve isotopically stationary conditions after feeding a ^13^C-labeled substrate.

In recent years, a novel method has been developed to determine metabolic flux using a time course of isotopic labeling data obtained from an isotopically transient state (Figure 1(d)) [15–17]. For the isotopically nonstationary MFA (INST-^13^C-MFA), an expanded metabolic model *M* is used to simulate isotopic labeling dynamics, taking into consideration the metabolite pool size in the cell:

(3) $$\begin{array}{c} {\text{MDV}}_{j,t=t_{k}}^{\text{sim}}=M\left(v,x^{\text{inp}},X,t_{k}\right), \end{array}$$

where *t*_*k*_ is the time of the *k*th sampling point. *X* is the vector of the pool sizes of all metabolites in the metabolic system. The formulation indicates that the intracellular pool sizes of intermediates in central metabolism *X* must be precisely determined for INST-^13^C-MFA [18, 19]. Time course analysis by rapid sampling techniques has also been performed in INST-^13^C-MFA to analyze the fast turnover of isotopic labeling enrichment in carbon central metabolism [20, 21]. Despite these technical challenges, INST-^13^C-MFA would be essential for the analysis of photoautotrophic organisms using CO_2_ as a carbon source. Metabolic flux cannot be determined by conventional ^13^C-MFA using ^13^CO_2_ as a carbon source, because all metabolites are uniformly labeled after reaching an isotopically stationary phase [22]. The methodology is also promising for the precise metabolic flux analysis of cells at a quasi-steady metabolic state (e.g., primary cells or microorganisms in stationary phase). In order to analyze a time course dataset produced by INST-^13^C-MFA, a software package with a graphical user interface has recently been reported (INCA [23]). In addition to these sophisticated tools, open source software packages such as OpenFLUX [14] for conventional ^13^C-MFA are also useful for facilitating the further development of INST-^13^C-MFA [24].

Here, a novel open source software package for INST-^13^C-MFA, OpenMebius (Open source software for Metabolic flux analysis), is reported. OpenMebius has been developed to perform INST-^13^C-MFA and conventional ^13^C-MFA using a user-defined metabolic model. A metabolic model *M* can be automatically generated from a metabolic pathway and a carbon transition network described in text or Microsoft Excel worksheet files. The metabolic flux distribution can be estimated by nonlinear fitting of the metabolic model to the isotopic labeling enrichment data.

## 2. Materials and Methods

### 2.1. Model Construction

OpenMebius is implemented in MATLAB (MathWorks, Natick, MA, USA) for the Windows platform. The software consists of two parts: automated model construction and metabolic flux estimation by nonlinear optimization. Functions for processing raw mass spectrum data and the determination of confidence intervals are also included. OpenMebius is designed for conventional ^13^C-MFA and INST-^13^C-MFA using mass spectrometry data. Isotopic labeling enrichment of metabolites is described by a mass distribution vector (MDV) [25]:

(4) $$\begin{array}{c} {\text{MDV}}_{j}=\left[\begin{array}{c} \begin{array}{c} m+0\\ m+1 \end{array}\\ \vdots \\ m+n \end{array}\right]\;\text{with}\;\;\overset{n}{\underset{i=0}{\sum }}m+i=1, \end{array}$$

where MDV_*j*_ is the vector of isotopic labeling enrichment of metabolite *j*. *m* + *i* indicates the relative abundance of a metabolite in which *i* carbons are labeled with ^13^C. To obtain the MDV_*j*_ of the carbon skeleton, mass spectrum data are corrected for the presence of naturally occurring isotopes using the correction matrix [26].

In conventional ^13^C-MFA, a metabolic model *M* is an algebraic equation used to generate MDV_*j*_^sim^ from the vector of metabolic flux (*v*) and the isotopic labeling pattern of a carbon source (*x* ^inp^), as shown in (1).

Since the metabolic flux is determined in cells at metabolic steady state, *v* follows the stoichiometric equation described by

(5) $$\begin{array}{c} Sv=0, \end{array}$$

where *S* is the stoichiometric matrix. In OpenMebius, *S* is constructed from a metabolic network described in the “Rxns” column in a user-defined configuration worksheet (Figure 1(b)), taking into consideration the fluxes for biomass syntheses and product excretion. MDV_*j*_^sim^ is calculated by the framework of elementary metabolite units (EMU) [27] using the carbon transition information described in the “carbon_transitions” column of the configuration worksheet (Figure 1(b)). In the framework, the carbon transition network is decomposed to cascade networks of EMUs depending on those carbon numbers. The cascade networks of the EMUs with *s*th carbon follow the EMU balance equation [27]:

(6) $$\begin{array}{c} A_{s}{\left(v\right)Z}_{s}=B_{s}\left(v\right)Y_{s}\left(x^{\text{inp}}\right). \end{array}$$

Here, each row in matrix *Z*_*s*_ is MDV of corresponding EMU. The matrix *Y*_*s*_(*x* ^inp^) includes EMUs of the carbon source or the smaller size EMUs. The element *a*_*s*_(*i*, *j*) in row *i* and column *j* of matrices *A*_*s*_(*v*) and the element *b*_*s*_(*i*, *j*) of matrix *B*_*s*_(*v*) are described, respectively, as follows:

(7) as(i,j) ={−sum  of  fluxes  consuming, ith  EMU  in  Zs,i=j,flux  to  ith  EMU  in  Zs  from  jth  EMU  in  Zs,i≠j,bs(i,j)=−flux  to  ith  EMU  in  Zs  from  jth  EMU  in  Ys.

In the case of INST-^13^C-MFA, the metabolic model *M* is expanded to describe a transition state of isotopic labeling (Figure 1(d)) by considering the dilution of isotopic labeling enrichment depending on the pool size of intermediates, as shown in (3), where *X* is a vector of the pool size of each metabolite that is constant under metabolic steady state. *t*_*k*_ is the time of the *k*th sampling point. In this study, instead of a direct description of the metabolic model *M*, time-dependent changes in the isotopic labeling enrichment of metabolite *j* are described by the differential equation as follows:

(8) dMDVj,t=tksimdt  =1Xj(∑i=1n(viinMDVi,t=tksim)−∑l=1m(vloutMDVj,t=tksim)),

where *v*_*i*_^in^ and *v*_*l*_^out^ represent the fluxes of the *i*th inflow reaction and the *l*th outflow reaction of metabolite *j*, respectively. The model is automatically constructed by “ConstEMUnetwork.m.” Detailed rules to describe a user-defined metabolic pathway and carbon transition network are provided on the project home page (http://www-shimizu.ist.osaka-u.ac.jp/hp/en/software/OpenMebius.html).* Euler*'s method is implemented to solve the ordinary differential equation (8) without adaptive step size control. Stiff equations can be resolved by carefully selecting the step size. The MDV_*j*,*t*=*t*_*k*__^sim^ are standardized for each step to prevent divergence. Moreover, no specific libraries were used to implement the algorithm for solving differential equations.

### 2.2. Metabolic Flux Estimation

**Figure 2:**
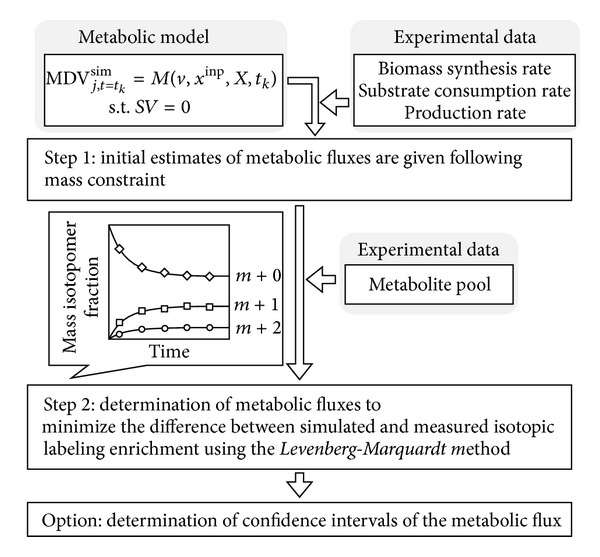
Procedure for metabolic flux estimation. Step 1: initial estimates of metabolic fluxes are given at random following the constraints of the mass balance, biomass synthetic rate, and substrate consumption and production rate. Step 2: metabolic fluxes are determined by minimizing the difference between simulated and measured isotopic labeling enrichment using the* Levenberg-Marquardt* method.

The procedure for estimating metabolic flux is shown in Figure 2. In Step 1, the initial flux distribution is given considering the rates of biomass synthesis, substrate consumption, and product excretion (Figure 2, Step 1). In Step 2, the metabolic flux vector *v* is optimized to minimize the covariance-weighted sum of squared difference (SSD) using the* Levenberg-Marquardt* method [28] (Figure 2, Step 2):

(9) D=MD^Vj,t=tk−M(v,xinp,X,tk),vopt=argminv∑k=1 Ns∑j=1N(DTCMD^Vj, t=tk−1D), s.t.  Sv=0.

Here, ${\text{M}\hat{\text{D}}\text{V}}_{j,{t=t}_{k}}$ is the vector of experimental data at *t* = *t*_*k*_. *N* is the total number of measured metabolites for data fitting. *Ns* is the total number of sampling points (*Ns* = 1 in the case of isotopically stationary), and $C_{{\text{M}\hat{\text{D}}\text{V}}_{j,{t=t}_{k}}}$ is the measurement covariance matrix with the measurement standard deviation located on the diagonal.

### 2.3. Calculation of Confidence Interval

Confidence intervals of estimated fluxes are determined by OpenMebius using the grid search method [29, 30]. The metabolic flux of reaction *r* is fixed to *v*_opt,*r*_ + *d* and the objective function is reoptimized. Here, *v*_opt,*r*_ is the optimized metabolic flux of reaction *r* and *d* is the perturbation level. The procedure is iterated with increased or decreased *d*. The range of fixed metabolic flux whose SSD is less than the threshold level is the confidence interval. The threshold level is determined by

(10) $$\begin{array}{c} \Phi_{\text{res},sr}\leq \Phi_{\text{res}}+\frac{\Phi_{\text{res}}}{n-p}F_{\alpha }\left(1,n-p\right), \end{array}$$

where Φ_res,*sr*_ is the minimized SSD with one fixed flux, Φ_res_ is the original minimized SSD, *n* is the number of independent data points used in the fitting, *p* is the degrees of freedom in the original flux fit, *F* is the *F*-distribution, and *α* is the confidence level.

## 3. Results and Discussion

### 3.1. Implementation

**Figure 3:**
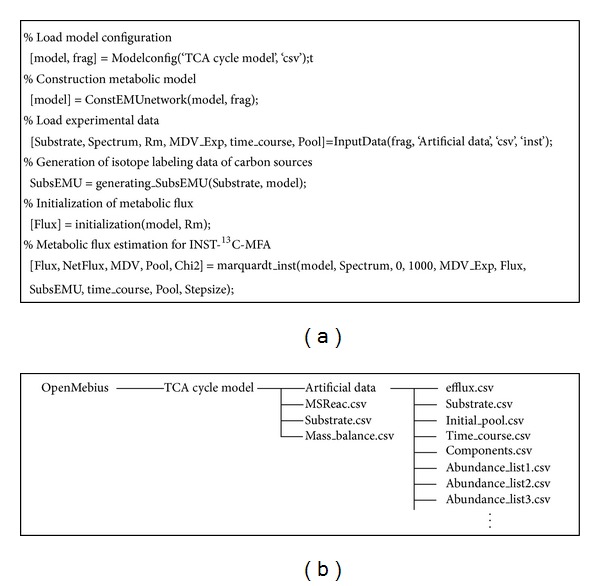
Representative Matlab code and structure of the metabolic model directory for isotopically nonstationary-^13^C-metabolic flux analysis using OpenMebius. (a) A metabolic model “model” is generated from information described in “TCA cycle model” directory by “Modelconfig” and “ConstEMUnetwork” functions. After loading time course mass spectrometry data in “Artificial data” directory, isotopic labeling data of carbon sources and an initial metabolic flux distribution are prepared. A metabolic flux distribution (“Flux”) is estimated by the “marquardt_inst” function using nonlinear optimization (Levenberg-Marquardt method). (b) Directory structure of the TCA cycle model. Configuration for simulating isotopic labeling, carbon source, and mass balance are described in MSReac.csv, Substrate.csv, and Mass_balance.csv, respectively. The simulated data directory includes a series of experimental data. Fluxes for biomass syntheses and product excretion, isotopic labeling information of substrate, metabolic concentrations of intracellular metabolite, and sampling times are described in efflux.csv, Substrate.csv, Initial_pool.csv, and Time_course.csv, respectively. Components.csv defines chemical elements in a mass fragment. A series of Abandance_list.csv are time course of isotopic labeling enrichment data. The “TCA cycle model” and online manual are available on web page (http://www-shimizu.ist.osaka-u.ac.jp/hp/en/software/OpenMebius.html).

OpenMebius is a toolbox for conventional ^13^C-MFA and INST-^13^C-MFA using mass spectrometry data implemented in MATLAB on the Windows platform.  Figure 3 shows a representative MATLAB code to perform INST-^13^C-MFA on a simplified TCA cycle model mentioned below. A metabolic model is generated by the “ConstEMUnetwork” function from user-defined metabolic network information described in text or Excel worksheet files. After loading related data, a metabolic flux distribution is estimated by the “marquardt_inst” function using a nonlinear optimization (*Levenberg-Marquardt* method). For a routine analysis, a batch execution of metabolic flux estimations is also supported. See Materials and Methods for detailed information.

### 3.2. Test Case of Isotopically Stationary MFA: Simplified TCA Cycle Model

**Figure 4:**
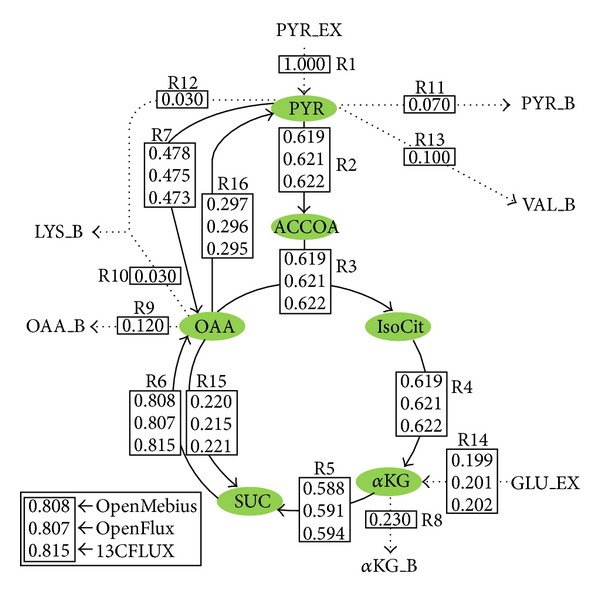
^13^C-MFA of simplified TCA model. Fluxes were calculated using OpenMebius, OpenFLUX, and 13CFLUX. Dotted lines indicate reactions whose metabolic fluxes are predetermined. Solid line arrows: intracellular metabolic reactions. Dotted line arrows: substrate consumption and biomass synthesis. Suffix: _B, biomass drain; _EX, exo-metabolites. Metabolites: PYR, pyruvate; ACCOA, acetyl-CoA; IsoCit, isocitrate; *α*KG, *α*-ketoglutarate; SUC, succinate; OAA, oxaloacetate; GLU, glutamate; LYS, lysine; VAL, valine.

The performance of OpenMebius for conventional ^13^C-MFA was tested with the simplified metabolic network used in the previous study [14] (Figure 4). The metabolic network consisted of the 16 reactions of the TCA cycle using pyruvate and glutamate as substrates described by Table 1. Among 16 metabolic fluxes, one influx (R1) and six effluxes (R8–R13) were predetermined. The metabolic model *M* was successfully constructed from the metabolic pathway and carbon transition networks. Here, the vector of experimental mass spectra (${\text{M}\hat{\text{D}}\text{V}}_{j}$) of valine, lysine, aspartate, and succinate was artificially created using the metabolic model, the flux distribution described in the previous research [14], and the isotopic labeling of pyruvate (mixture of 50% 1-^13^C and 50% U-^13^C) and glutamate (100% 1-^13^C). Considering the simulated data as the measured MDV, the metabolic flux distribution was determined by the conventional ^13^C-MFA function of OpenMebius. The estimated flux distribution was essentially identical to that of simulated distribution, which was consistent with the results of 13CFLUX [8] and OpenFLUX [14] (Figure 4). The total computation time was 6 seconds for 10 cycles of optimization (Intel Core i7 2.80 GHz), which was the same as in OpenFLUX.

### 3.3. Test Case of Isotopically Nonstationary MFA: Simplified TCA Cycle Model

**Figure 5:**
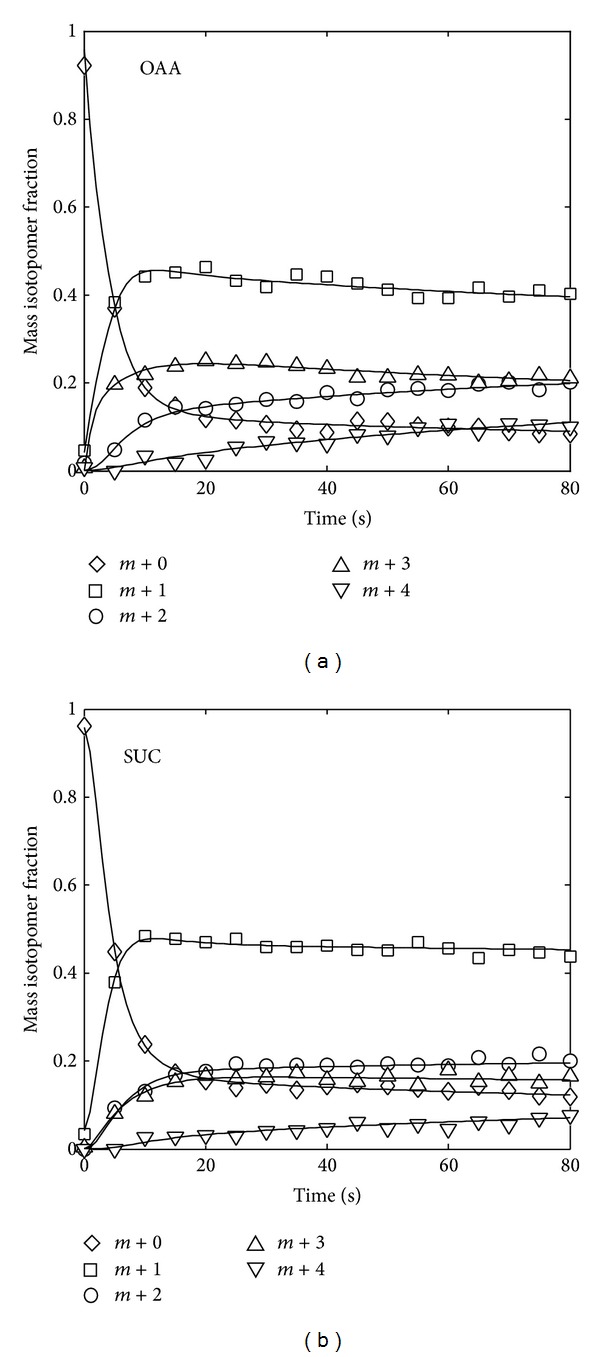
Dynamics of isotopic labeling enrichment. Time course of fitted (solid lines) and simulated (symbols) isotopic labelling enrichment is shown. OAA, oxaloacetate; SUC, succinate.

To simulate an isotopic labeling experiment during an isotopically nonstationary period, the pool size information of six intermediates was arbitrarily added to the above TCA metabolic network. A metabolic model for INST-^13^C-MFA was successfully constructed by OpenMebius. To prepare simulated experimental data, time course data of isotopic labeling dynamics of oxaloacetate and succinate were created using the differential equation (8) combined with the pool size information (*X*). The current version of OpenMebius uses the pool size information (*X*) as constant values, although *X* should be estimated with an optimization procedure since the pool size data are less reliable than isotopic labeling measurements. That function will be supported in a future version of OpenMebius. The flux distribution (*v*) and isotopic labeling patterns of substrate (*x* ^inp^) were identical to those of the previous section. The MDVs of oxaloacetate and succinate were sampled 17 times at 5-second intervals* in silico*, to which Gaussian noise (1%) was added to imitate actual measurements. Considering the simulated data as measured MDVs (${\text{M}\hat{\text{D}}\text{V}}_{j,{t=t}_{k}}$), the metabolic flux distribution was estimated using OpenMebius. The step size was set to 0.01 seconds to compute the simulated MDVs. Although only two intracellular metabolites were used for data fitting, the fitted isotopic labeling dynamics and a flux distribution were consistent with the simulated data (Figure 5). The total computational time for one cycle of optimization was around 10 minutes (Intel Core i7 2.80 GHz).

**Figure 6:**
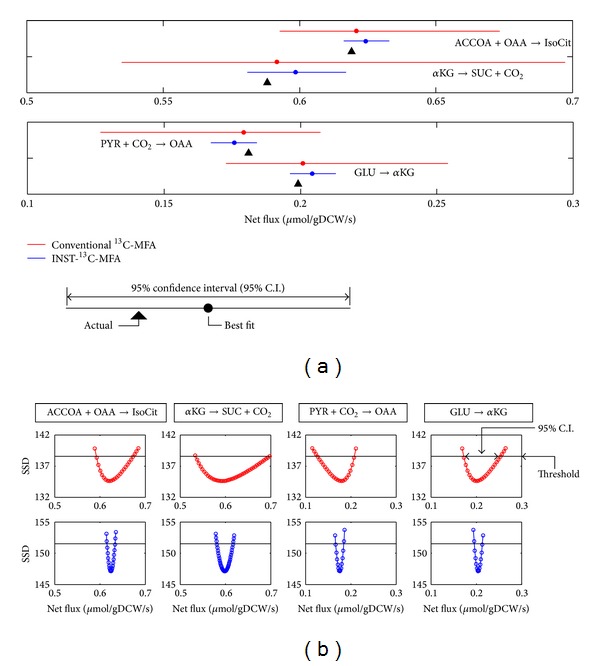
Comparison of confidence intervals between INST-^13^C-MFA and conventional ^13^C-MFA. (a) 95% confidence intervals of four representative fluxes were compared between conventional ^13^C-MFA (red) and INST-^13^C-MFA (blue). Black triangles indicate the actual values. (b) Shapes of reoptimized sum of squared difference (SSD) determined by the grid search. The red and blue lines show the results of conventional ^13^C-MFA and INST-^13^C-MFA, respectively. The horizontal lines represent the threshold value for the 95% confidence intervals.

For a performance comparison between conventional ^13^C-MFA and INST-^13^C-MFA, the 95% confidence intervals of four representative reactions were determined by the grid search method (Figure 6(a)). For INST-^13^C-MFA, confidence intervals were estimated using the simulated data with the 17 time points prepared above. In the case of conventional ^13^C-MFA, a novel simulated dataset was prepared by the following procedure. From the MDV_*j*_^sim^ of oxaloacetate and succinate calculated using (1), 17 sets of simulated mass spectra (${\text{M}\hat{\text{D}}\text{V}}_{j}$) data were produced with the addition of Gaussian noise (1%). While an identical number of data points was used, the confidence intervals determined by INST-^13^C-MFA were approximately 22% that of conventional ^13^C-MFA (Figure 6(a)). The sharply curved parabolas were observed for INST-^13^C-MFA, suggesting that the time course MDV data includes information for a more precise estimation of metabolic flux (Figure 6(b)). These results suggest that INST-^13^C-MFA could be a reliable method to determine* in vivo* metabolic flux with narrow confidence intervals.

### 3.4. Test Case of Isotopically Nonstationary MFA:* Escherichia coli* Model

**Figure 7:**
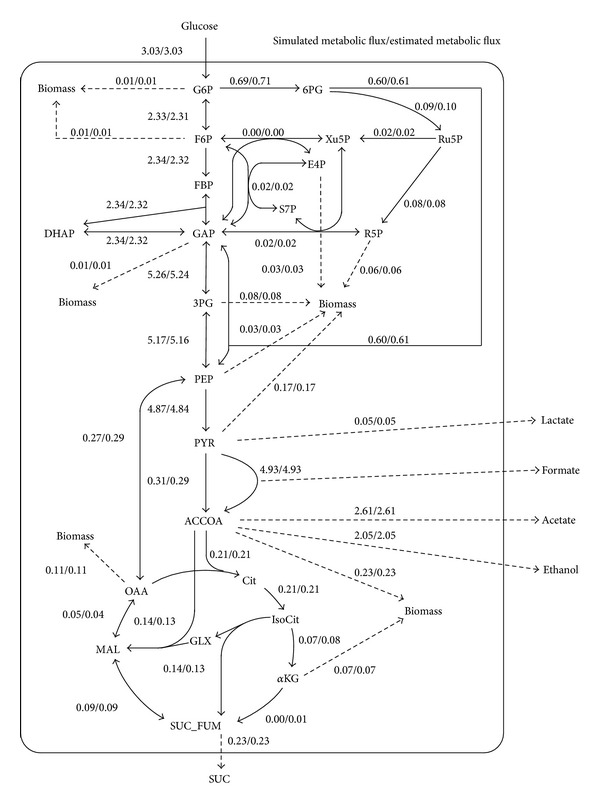
Metabolic network of* Escherichia coli*. Solid lines and dotted lines indicate intracellular metabolic reactions and product excretion, respectively. The arrows represent directions of the reactions. Simulated metabolic flux and estimated metabolic flux are shown left and right, respectively. All fluxes are given as absolute values (*μ*mol gDCW^−1^ s^−1^). Abbreviations are shown in the Abbreviations section.

INST-^13^C-MFA was also performed using simulated data produced from the central metabolic model of* E. coli* with 54 reactions and 22 intermediates. A simulated experimental dataset was prepared based on the literature-reported metabolic flux distribution and metabolite pool size data [31]. Pool sizes of unmeasured metabolites (GAP, PYR, Xu5P, E4P, IsoCit, *α*KG, and glyoxylate) were arbitrarily set at 0.1 *μ*mol gDCW^−1^. Simulated MDVs were sampled 11 times at 1-second intervals using 100% [1-^13^C] glucose as a carbon source. Considering the simulated dataset as experimental data, metabolic fluxes were estimated using the INST-^13^C-MFA function of OpenMebius. The step size was set to 0.001 seconds to compute the simulated MDVs. Although the computation time took 7 h 42 min (Intel Xeon X5670 2.93 GHz), the estimated flux distribution was essentially identical to that of the simulated data (Figure 7). The result indicates that OpenMebius could deal with INST-^13^C-MFA using a realistic metabolic model of* E. coli*.

## 4. Conclusions

OpenMebius is the first open source software for metabolic flux analyses under both isotopically stationary and nonstationary conditions. The software supports the automatic construction of a metabolic model for INST-^13^C-MFA from a user-defined metabolic network. Analysis using simulated data demonstrated not only the utility of OpenMebius for INST-^13^C-MFA, but also its potential for use in metabolic flux analysis with reduced confidence intervals. OpenMebius provides an essential bioinformatics tool for INST-^13^C-MFA to analyze metabolic flux in cells with slower metabolism (i.e., mammalian) [17] and cultivation with single carbon substrates (i.e., cyanobacteria) [15].

## Figures and Tables

**Table 1:** Configuration of TCA cycle model.

FluxID	Rxns	Net flux	Carbon transitions
R1	Subs_PYR_EX → PYR	0	abc → abc
R2	PYR → ACCOA + Ind_CO2	0	abc → bc + a
R3	ACCOA + OAA → IsoCit	0	ab + cdef → fedbac
R4	IsoCit → AKG + Ind_CO2	0	abcdef → abcde + f
R5	AKG → Sym_SUC + Ind_CO2	0	abcde → abcd + e
R6	Sym_SUC → OAA	1	abcd → abcd
R7	PYR + Ind_CO2 → OAA	2	abc + d → abcd
R8	AKG → [AKG_B]	0	
R9	OAA → [OAA_B]	0	
R10	OAA → [LYS_B]	0	
R11	PYR → [PYR_B]	0	
R12	PYR → [LYS_B]	0	
R13	PYR → [VAL_B]	0	
R14	Subs_GLU_EX → AKG	0	abcde → abcde
R15	OAA → Sym_SUC	1	abcd → abcd
R16	OAA → PYR + Ind_CO2	2	abcd → abc + d

## Abbreviations

MDV: Mass distribution vector
^13^C-MFA: ^13^C-based metabolic flux analysis
INST-^13^C-MFA: Isotopically nonstationary ^13^C metabolic flux analysis
*α*KG: *α*-Ketoglutarate
ACCOA: Acetyl-CoA
Cit: Citrate
DHAP: Dihydroxyacetone phosphate
E4P: Erythrose-4-phosphate
F6P: Fructose-6-phosphate
FBP: Fructose-1,6-bisphosphate
FUM: Fumarate
G6P: Glucose-6-phosphate
GAP: Glyceraldehyde-3-phosphate
GLX: Glyoxylate
IsoCit: Isocitrate
MAL: Malate
OAA: Oxaloacetate
PEP: Phosphoenolpyruvate
6PG: 6-Phosphoglycerate
3PG: 3-Phosphoglycerate
PYR: Pyruvate
R5P: Ribose-5-phosphate
Ru5P: Ribulose-5-phosphate
S7P: Sedoheptulose-7-phosphate
SUC: Succinate
SUC_FUM: Sum of metabolite pool of succinate and fumarate
Xu5P: Xylulose-5-phosphate
VAL: Valine
LYS: Lysine.

## References

[1] Sang YL, Lee D-Y, Tae YK (2005). Systems biotechnology for strain improvement. *Trends in Biotechnology*.

[2] Moxley JF, Jewett MC, Antoniewicz MR (2009). Linking high-resolution metabolic flux phenotypes and transcriptional regulation in yeast modulated by the global regulator Gcn4p. *Proceedings of the National Academy of Sciences of the United States of America*.

[3] Duckwall CS, Murphy TA, Young JD (2013). Mapping cancer cell metabolism with ^13^C flux analysis: recent progress and future challenges. *Journal of Carcinogenesis*.

[4] Frick O, Wittmann C (2005). Characterization of the metabolic shift between oxidative and fermentative growth in Saccharomyces cerevisiae by comparative ^13^C flux analysis. *Microbial Cell Factories*.

[5] Toya Y, Ishii N, Nakahigashi K (2010). ^13^C-Metabolic flux analysis for batch culture of *Escherichia coli* and its pyk and pgi gene knockout mutants based on mass isotopomer distribution of intracellular metabolites. *Biotechnology Progress*.

[6] Mori E, Furusawa C, Kajihata S, Shirai T, Shimizu H (2011). Evaluating^13^C enrichment data of free amino acids for precise metabolic flux analysis. *Biotechnology Journal*.

[7] Shirai T, Fujimura K, Furusawa C, Nagahisa K, Shioya S, Shimizu H (2007). Study on roles of anaplerotic pathways in glutamate overproduction of *Corynebacterium glutamicum* by metabolic flux analysis. *Microbial Cell Factories*.

[8] Wiechert W, Möllney M, Petersen S, De Graaf AA (2001). A universal framework for ^13^C metabolic flux analysis. *Metabolic Engineering*.

[9] Weitzel M, Nöh K, Dalman T, Niedenführ S, Stute B, Wiechert W (2013). ^13^CFLUX2—high-performance software suite for ^13^C-metabolic flux analysis. *Bioinformatics*.

[10] Cvijovic M, Olivares-Hernandez R, Agren R (2010). BioMet Toolbox: genome-wide analysis of metabolism. *Nucleic Acids Research*.

[11] Yoo H, Antoniewicz MR, Stephanopoulos G, Kelleher JK (2008). Quantifying reductive carboxylation flux of glutamine to lipid in a brown adipocyte cell line. *Journal of Biological Chemistry*.

[12] Srour O, Young JD, Eldar YC (2011). Fluxomers: a new approach for ^13^C metabolic flux analysis. *BMC Systems Biology*.

[13] Sokol S, Millard P, Portais J-C (2012). Influx_s: increasing numerical stability and precision for metabolic flux analysis in isotope labelling experiments. *Bioinformatics*.

[14] Quek L-E, Wittmann C, Nielsen LK, Krömer JO (2009). OpenFLUX: efficient modelling software for ^13^C-based metabolic flux analysis. *Microbial Cell Factories*.

[15] Young JD, Shastri AA, Stephanopoulos G, Morgan JA (2011). Mapping photoautotrophic metabolism with isotopically nonstationary^13^C flux analysis. *Metabolic Engineering*.

[16] Schaub J, Mauch K, Reuss M (2008). Metabolic flux analysis in *Escherichia coli* by integrating isotopic dynamic and isotopic stationary ^13^C labeling data. *Biotechnology and Bioengineering*.

[17] Murphy TA, Dang CV, Young JD (2013). Isotopically nonstationary ^13^C flux analysis of Myc-induced metabolic reprogramming in B-cells. *Metabolic Engineering*.

[18] Zhou B, Xiao JF, Tuli L, Ressom HW (2012). LC-MS-based metabolomics. *Molecular BioSystems*.

[19] Vuckovic D (2012). Current trends and challenges in sample preparation for global metabolomics using liquid chromatography-mass spectrometry. *Analytical and Bioanalytical Chemistry*.

[20] Schädel F, Franco-Lara E (2009). Rapid sampling devices for metabolic engineering applications. *Applied Microbiology and Biotechnology*.

[21] van Gulik WM (2010). Fast sampling for quantitative microbial metabolomics. *Current Opinion in Biotechnology*.

[22] Shastri AA, Morgan JA (2007). A transient isotopic labeling methodology for ^13^C metabolic flux analysis of photoautotrophic microorganisms. *Phytochemistry*.

[23] Young JD (2014). INCA: a computational platform for isotopically non-stationary metabolic flux analysis. *Bioinformatics*.

[24] Wiechert W, Nöh K (2013). Isotopically non-stationary metabolic flux analysis: complex yet highly informative. *Current Opinion in Biotechnology*.

[25] Wittmann C, Heinzle E (1999). Mass spectrometry for metabolic flux analysis. *Biotechnology and Bioengineering*.

[26] Van Winden WA, Wittmann C, Heinzle E, Heijnen JJ (2002). Correcting mass isotopomer distributions for naturally occurring isotopes. *Biotechnology and Bioengineering*.

[27] Antoniewicz MR, Kelleher JK, Stephanopoulos G (2007). Elementary metabolite units (EMU): a novel framework for modeling isotopic distributions. *Metabolic Engineering*.

[28] Press WH, Flannery BP, Teukolsky SA, Vetterling WT (2007). *Numerical Recipes in C: the Art of Scientific Computing*.

[29] Antoniewicz MR, Kelleher JK, Stephanopoulos G (2006). Determination of confidence intervals of metabolic fluxes estimated from stable isotope measurements. *Metabolic Engineering*.

[30] Costenoble R, Müller D, Barl T (2007). ^13^C-Labeled metabolic flux analysis of a fed-batch culture of elutriated Saccharomyces cerevisiae. *FEMS Yeast Research*.

[31] Toya Y, Nakahigashi K, Tomita M, Shimizu K (2012). Metabolic regulation analysis of wild-type and *arcA* mutant *Escherichia coli* under nitrate conditions using different levels of omics data. *Molecular BioSystems*.

